# Integrated digital inverters based on two-dimensional anisotropic ReS_2_ field-effect transistors

**DOI:** 10.1038/ncomms7991

**Published:** 2015-05-07

**Authors:** Erfu Liu, Yajun Fu, Yaojia Wang, Yanqing Feng, Huimei Liu, Xiangang Wan, Wei Zhou, Baigeng Wang, Lubin Shao, Ching-Hwa Ho, Ying-Sheng Huang, Zhengyi Cao, Laiguo Wang, Aidong Li, Junwen Zeng, Fengqi Song, Xinran Wang, Yi Shi, Hongtao Yuan, Harold Y. Hwang, Yi Cui, Feng Miao, Dingyu Xing

**Affiliations:** 1 National Laboratory of Solid State Microstructures, School of Physics, Collaborative Innovation Center of Advanced Microstructures, Nanjing University, Nanjing 210093, China; 2 Geballe Laboratory for Advanced Materials, Stanford University, Stanford, California 94305, USA; 3 Stanford Institute for Materials and Energy Sciences, SLAC National Accelerator Laboratory, Menlo Park, California 94025, USA.; 4 Graduate School of Applied Science and Technology, National Taiwan University of Science and Technology, Taipei 106, Taiwan; 5 Department of Electronic and Computer Engineering, National Taiwan University of Science and Technology, Taipei 106, Taiwan; 6 Department of Materials Science and Engineering, College of Engineering and Applied Sciences, Nanjing University, Nanjing 210093, China; 7 School of Electronic Science and Engineering, Nanjing University, Nanjing 210093, China

## Abstract

Semiconducting two-dimensional transition metal dichalcogenides are emerging as top candidates for post-silicon electronics. While most of them exhibit isotropic behaviour, lowering the lattice symmetry could induce anisotropic properties, which are both scientifically interesting and potentially useful. Here we present atomically thin rhenium disulfide (ReS_2_) flakes with unique distorted 1T structure, which exhibit in-plane anisotropic properties. We fabricated monolayer and few-layer ReS_2_ field-effect transistors, which exhibit competitive performance with large current on/off ratios (∼10^7^) and low subthreshold swings (100 mV per decade). The observed anisotropic ratio along two principle axes reaches 3.1, which is the highest among all known two-dimensional semiconducting materials. Furthermore, we successfully demonstrated an integrated digital inverter with good performance by utilizing two ReS_2_ anisotropic field-effect transistors, suggesting the promising implementation of large-scale two-dimensional logic circuits. Our results underscore the unique properties of two-dimensional semiconducting materials with low crystal symmetry for future electronic applications.

## Abstract (web summary)

Many two-dimensional materials exhibit isotropic properties, but anisotropy can extend the functionality of future devices. Here, the authors fabricate field-effect transistors from single and few-layer rhenium disulfide and observe an anisotropic ratio of three to one along the two principle axes

## Graphical abstract

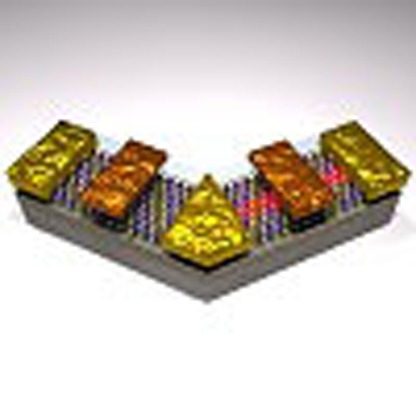

The scaling down of conventional metal-oxide-semiconductor FETs is approaching the limit of miniaturization due to severe short-channel effects^1^. One approach to solve this problem is searching for atomically thin semiconductors, such as semiconducting two-dimensional (2D) materials^2^,^3^,^4^,^5^,^6^. Among the potential candidates, the family of transition metal dichalcogenides (TMDs)^7^,^8^ (MX_2_, where M denotes a transition metal and X denotes a chalcogen) has attracted the most attention due to both its rich physics and tremendous potential for application. They are expected to exhibit a wide range of electronic structures and exotic transport properties arising from the various electron configurations of transition metals, such as superconductivity^9^,^10^,^11^, half-metallic magnetism^12^ and charge density waves^13^,^14^,^15^.

Lattice structure and symmetry are vital in determining materials' fundamental properties. Most studied 2D TMDs exhibit isotropic behaviour due to high lattice symmetry; however, lowering the symmetry in TMDs could induce interesting anisotropic properties of both scientific and technologic importance. For example, in WTe_2_, which is a semi-metallic TMD with a distorted lattice structure, a large non-saturating magnetoresistance effect along a principle axis (the direction of W chain formation) has been recently reported and has attracted considerable attention^16^. In contrast, semiconducting 2D TMDs with low symmetry have remained unexplored.

As the last stable element discovered in nature, the transition metal rhenium (Re) exhibits a wide range of oxidation states and crucial applications, such as superalloys and catalytic reforming. Its sulfide rhenium disulfide (ReS_2_) is a semiconducting TMD that was recently discovered to have weak interlayer coupling and a unique distorted 1T structure^17^. As shown by the side view and top view of the monolayer ReS_2_ crystal structure in Fig. 1a, it consists of two hexagonal planes of S atoms (yellow) and an intercalated hexagonal plane of Re atoms (blue) bound with S atoms forming chains of parallelogram-shaped Re4 clusters. As highlighted by the red arrows in the top view, there are two principle axes, the *b* and *a* axes, which correspond to the shortest and second-shortest axes in the basal plane. They are 61.03° or 118.97° apart, and the *b* axis corresponds to the direction of the Re–Re atomic chain formed. Thus, ReS_2_ offers an ideal material candidate for studying semiconducting 2D TMDs with low symmetry.

In this work, we systematically investigate the in-plane anisotropic properties of atomically thin ReS_2_. In addition to highly competitive FET performance, we also observe a large anisotropic ratio along its two principle axes, which is the highest among all experimentally investigated 2D layered materials. We also successfully demonstrate a digital inverter with good performance by integrating two anisotropic ReS_2_ FET devices. Our results suggest that the lattice orientation can be used as an alternative design variable to tune device properties and optimize circuit performance in future 2D integrated circuits based on the anisotropic semiconducting materials.

## Results

### Material characterizations and *ab initio* calculations

**Figure 1:**
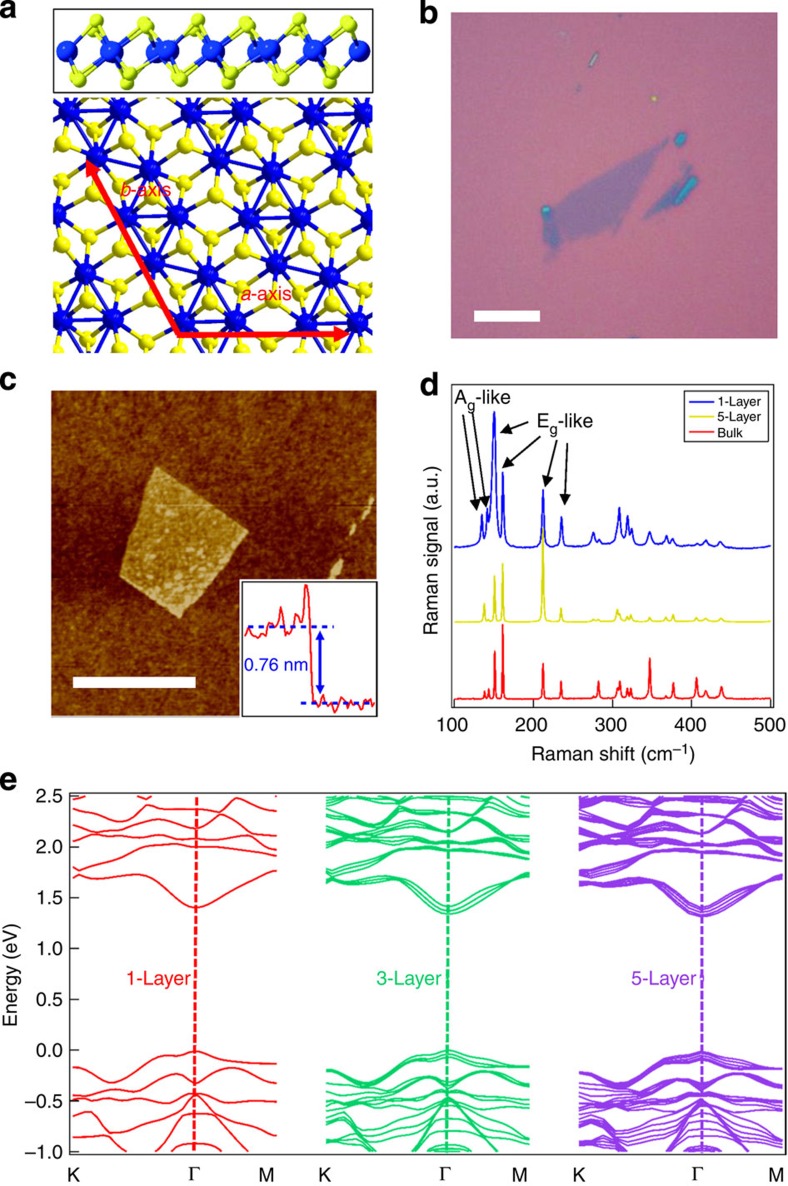
Characterization and band structure of thin-layer ReS_2_. (**a**) Crystal structure of monolayer ReS_2_ with a side view in the top panel and a top view in the bottom panel. Both directions of *a* and *b* axes are denoted by red arrows. (**b**) Optical image of a monolayer ReS_2_ flake. Scale bar, 10 μm. (**c**) AFM image of a monolayer ReS_2_ flake. Scale bar, 1 μm. Inset: height profile along the blue line indicating a single layer. (**d**) Micro Raman experimental results performed on monolayer, five-layer and bulk ReS_2_. Six labelled Raman modes include two low frequency A_g_-like modes corresponding to the out-of-plane vibrations of Re atoms and four E_g_-like modes corresponding to the in-plane vibrations of Re atoms. The rest 12 higher frequency Raman modes are vibrations mainly from lighter S atoms. (**e**) Band structure of monolayer, trilayer and five-layer ReS_2_ by *ab initio* calculations indicating band gaps of 1.44, 1.40 and 1.35 eV, respectively. AFM, atomic force microscope.

Figure 1b,c shows typical optical microscopy and atomic force microscope images of a monolayer ReS_2_ film (see Methods for the material details), respectively. The inset of Fig. 1c shows that the thickness of the monolayer film is ∼0.8 nm. In this study, we focus on monolayer and few-layer ReS_2_, with thicknesses ranging from 0.8 to 5 nm (1–7 layers with interlayer spacing of ∼0.7 nm). Micro Raman scattering experiments were performed on monolayer, five-layer and bulk ReS_2_. Due to the low symmetry of ReS_2_, 18 Raman modes were observed as shown in Fig. 1d. The peak positions of our three samples are close to each other, same to the reported results^17^. In Fig. 1d, we labelled two low frequency A_g_-like modes (located at 136.8 and 144.5 cm^−1^) corresponding to the out-of-plane vibrations of Re atoms and four E_g_-like modes (located at 153.6, 163.4, 218.2 and 238.1 cm^−1^) corresponding to the in-plane vibrations of Re atoms. The rest 12 higher frequency Raman modes are vibrations mainly from lighter S atoms^18^. The peak intensity ratio could be dependent on the polarization due to the low lattice symmetry^19^. The band structures of monolayer, trilayer and five-layer ReS_2_ calculated by an *ab initio* method are shown in Fig. 1e, indicating the nature of a direct band gap semiconductor without the indirect-to-direct band gap transition observed in other TMDs^3^. The overall band topology does not significantly change, with only a minor band gap shortening from monolayer (1.44 eV), trilayer (1.4 eV) to five-layer (1.35 eV).

### FET performances

**Figure 2:**
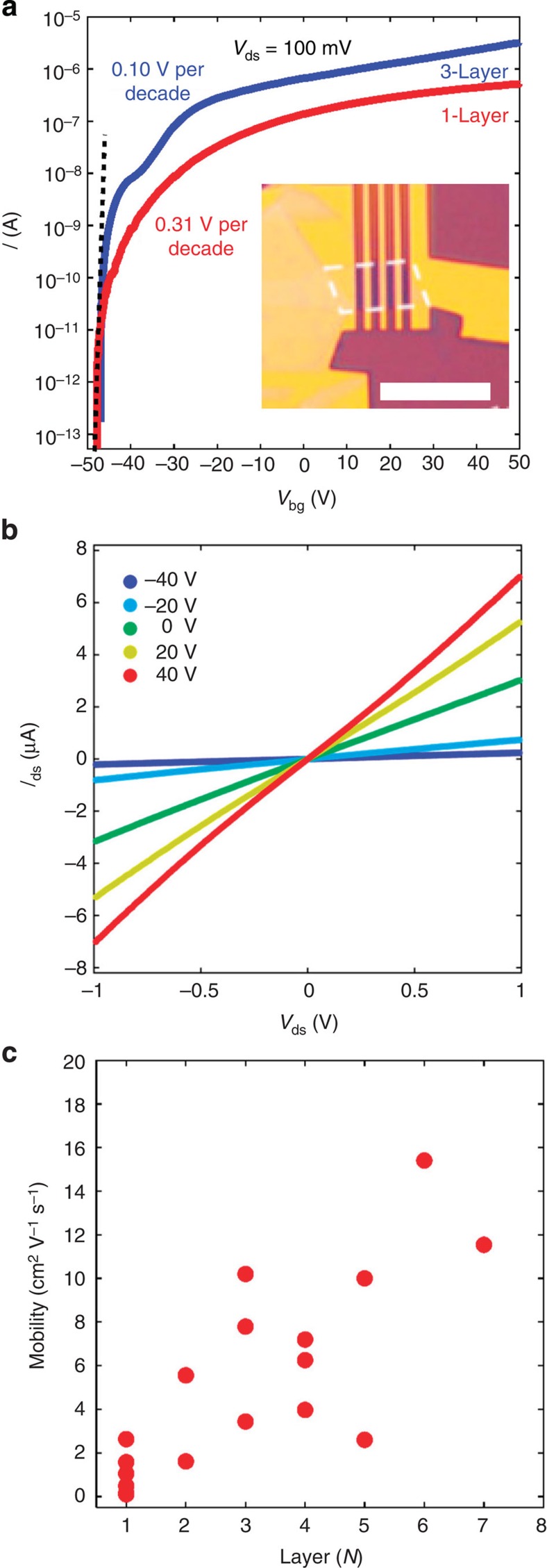
ReS_2_ field-effect transistor devices. (**a**) Transfer curves of monolayer (red) and trilayer (blue) ReS_2_ FET devices. *V*_ds_ is fixed to 100 mV. The on/off ratio is ∼10^7^ for the monolayer device and 10^7^ for the seven-layer device. The subthreshold swings are 310 mV per decade (monolayer) and 100 mV per decade (trilayer), respectively. Inset: optical image of a typical monolayer ReS_2_ FET device. Scale bar, 5 μm. (**b**) *I*_ds_–*V*_ds_ curves of a monolayer ReS_2_ FET at different *V*_bg_, with linear dependence indicating the ohmic contact. (**c**) The dependence of device mobility on the number of layers. In general, the mobility increases monotonically with the number of layers with some scattering.

We first examine the device performances of monolayer and few-layer ReS_2_ FET devices (see Supplementary Note 1 for details). The inset of Fig. 2a presents the optical image of a typical monolayer device. The FET transfer curve was obtained by monitoring the source–drain current *I*_ds_ while sweeping the back gate voltage *V*_bg_. A fixed 100-mV source–drain bias voltage *V*_sd_ was applied across the channel during all of the measurements. We first measured a couple of monolayer ReS_2_ devices, all of which behaved as excellent n-type FET devices with the back gate swept between −50 and +50 V. As shown in Fig. 2a, the current on/off ratio reached 10^7^, which meets the requirement of many applications, such as digital logic computation. When the back gate voltage reached +50 V, the *I*_ds_ approached saturation. Few-layer ReS_2_ transistor devices, ranging from bilayer to seven-layer, were studied as well. Similar n-type FET behaviour but a slightly larger on/off ratio was observed. The results from a trilayer device with the same back gate sweep range are shown in Fig. 2a for comparison. We note that all of the above measurements were carried out under vacuum (∼10^−5^ mbar) at room temperature, and ambipolar behaviour can be observed by introducing the ionic liquid gating technique (see Supplementary Note 2 for details). On the basis of the transfer curves, we can also measure the subthreshold swing. For the monolayer ReS_2_ FET device shown in Fig. 2a, the subthreshold swing is ∼310 mV per decade. The subthreshold swing becomes steeper for thicker flakes, reaching ∼100 mV per decade for the trilayer device shown in the same figure. The measured subthreshold swing values approach those measured in top-gated MoS_2_ transistors^7^ and outperform black phosphorus devices^5^. This can be further improved by increasing the gate capacitance after introducing thinner high-κ dielectric materials.

The monolayer and few-layer ReS_2_ FET devices (with Ti/Au electrodes) have also shown good contact behaviour. As shown in Fig. 2b, for a typical monolayer device, the source–drain current *I*_ds_ varies linearly with the bias *V*_ds_ in the ±1 V range at different back gate voltages (*V*_bg_=−40, −20, 0, 20 and 40 V, respectively), indicating ohmic contact behaviour in the n-doped regime under consideration (see Supplementary Note 3 for more details).

We further extracted the mobility of all of the FET devices we measured and studied its dependence on the number of layers of the ReS_2_. The mobility was determined by taking the steepest slope in the two-terminal *I*_ds_–*V*_bg_ curves. The results for 17 devices (from monolayer to seven-layer) are plotted in Fig. 2c. Similar to the reported results based on MoS_2_ thin flakes^20^,^21^, the device mobility generally increases with an increasing number of layers, implying that the electrons likely independently transport through different layers in thin ReS_2_ flakes. The mobility of a monolayer device varies between 0.1 and 2.6 cm^2^ V^−1^ s^−1^, and the highest mobility value obtained is 15.4 cm^2^ V^−1^ s^−1^ for a six-layer device. These values were measured without any material treatment or device optimization. With further improvement in crystal quality, post-fabrication treatment^22^ and dielectric engineering^7^,^22^, considerable enhancement of mobility to approach application requirements should be expected. Here the scattering of mobility values for flakes with the same number of layers could be induced by device quality variations or the anisotropic property, as will be discussed below.

### Anisotropic properties of ReS_2_

**Figure 3:**
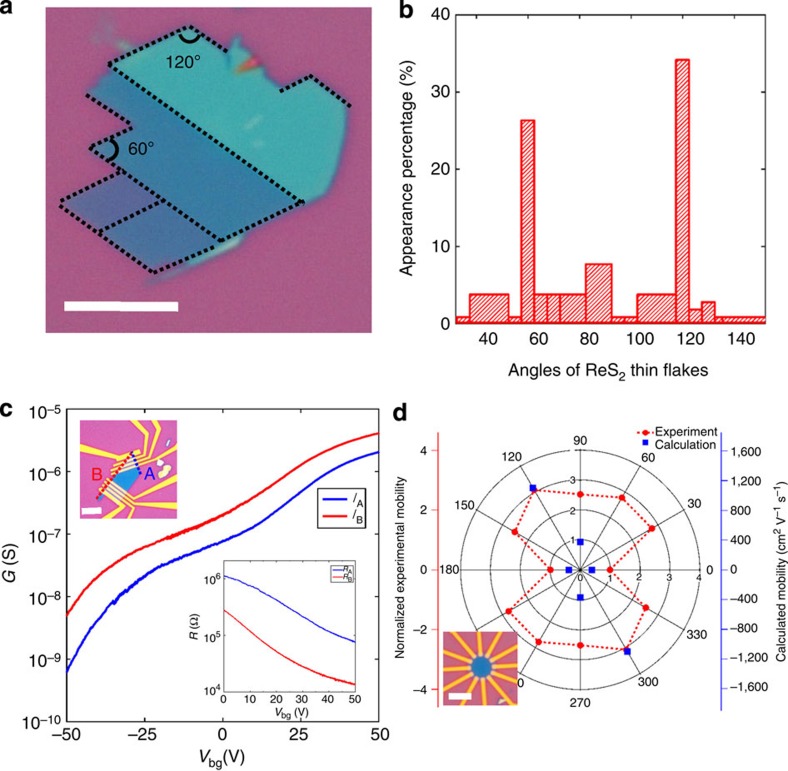
Anisotropic properties of ReS_2_. (**a**) Optical image of a typical thin ReS_2_ flake with a quadrilateral shape. Scale bar, 5 μm. (**b**) The statistics of inner angles for over 20 thin ReS_2_ flakes, showing the greatest prevalence for 60° and 120°. (**c**) Transfer curves of anisotropic ReS_2_ FETs along two sides (A and B direction) of a quadrilateral-shaped five-layer flake (with an inner angle of 60° or 120°). Top inset: optical image of the devices. Scale bar, 10 μm. Low inset: the 4-probe resistance of the same devices with *V*_bg_ varying between 0 and 60 V. (**d**) Normalized field-effect mobility of a six-layer device along 12 directions evenly spaced at 30° apart plotted in polar coordinates (red dots with left axis). The direction with the lowest mobility was set to be the 0° (or 180°) reference. The optical image of the device is shown in the inset. The calculated mobility of monolayer ReS_2_ along three orientations (*a* axis, *b* axis and perpendicular to the *a* axis) is plotted in the same graph (blue dots with right axis) for comparison. The lowest mobility (*a* axis) direction was set to be the 0° (or 180°) reference as well.

We now focus on the in-plane anisotropic properties of monolayer and few-layer ReS_2_ flakes induced by low lattice symmetry. As described in Fig. 1a, the *a* and *b* axes are the two directions with the shortest axes in the basal plane, thus differentiating them from other lattice orientations. Such a feature is often associated with the in-plane anisotropic properties of structural stiffness and electronic transport. Interestingly, during our experiments, exfoliated thin ReS_2_ flakes commonly appear in a quadrilateral shape with inner angles of ∼60° or 120°, as shown by a typical flake in Fig. 3a. Further results of all inner angles measured on over 20 thin flakes is shown in Fig. 3b, with >60% of the specimens showing these two angles. The inner angles of 60° and 120° match the angles between the *a* and *b* axes (118.97° or 61.03°) with high accuracy. This can be readily explained by the fact that the breaking strength is the weakest along these two axes, which are the two most strongly bonded orientations^17^,^23^, and suggests that two sides of the quadrilateral shape with 60° or 120° inner angles correspond to the *a* and *b* axes, respectively.

The in-plane stiffness anisotropy-induced quadrilateral shape makes it feasible to determine the two axes of ReS_2_ flakes via convenient transport measurements instead of other highly sophisticated tools, such as scanning tunnelling microscopy. Early studies on bulk materials reported that the *b* axis is more conductive than other crystalline orientations^24^,^25^. We then patterned the electrodes to be perpendicular to two sides (A and B directions) of a quadrilateral-shaped five-layer ReS_2_ with a 60° inner angle (as shown in the top inset of Fig. 3c). The transfer curves in two directions are shown in Fig. 3c, and anisotropic FET behaviour was observed. The B direction appears to be noticeably more conductive than the A direction, suggesting that the A and B directions correspond to the *a* and *b* axes, respectively. The conductance ratio of the two directions is gate dependent, with values of ∼8.2 when *V*_bg_=−50 V and ∼2 when *V*_bg_=50 V. To evaluate the influence of contact resistance, we performed four-terminal measurements (see Supplementary Note 3 for more details). The four-terminal results of the same device are shown in the lower inset of Fig. 3c, indicating that the anisotropy arises from the intrinsic properties of ReS_2_.

We further systematically studied the anisotropic properties of thin ReS_2_ flakes through angle-resolved transport measurement. As shown in the inset of Fig. 3d, a six-layer device was fabricated with 12 electrodes (5 nm Ti/50 nm Au) evenly spaced at 30° apart. We measured the transfer curves of each pair of diagonally positioned electrodes separated by 4.5 μm at 180° apart and extracted the renormalized field-effect mobility of each direction, with the results plotted in Fig. 3d (red dots) in polar coordinates. Measurements on each pair of electrodes lead to two data points that are 180° apart by swapping source–drain current directions. Here we define the direction with the lowest mobility to be the 0° (or 180°) reference. The field-effect mobility is highly angle dependent, with the largest value in the direction of 120° (or 300°), which is 60° from the direction with the lowest value. The anisotropic ratio of mobility *μ*_max_/*μ*_min_ is ∼3.1, which is noticeably larger than that reported in other 2D anisotropic materials, such as 1.8 for thin-layer black phosphorus^26^.

To fully understand our observations, we calculated the effective mass and mobility of monolayer ReS_2_ along three crystalline orientations (*a* axis, *b* axis and perpendicular to the *a* axis) using an *ab initio* technique (see Supplementary Note 4 for details), with the results plotted in the same graph (blue dots with right axis). Here we set the direction with the lowest mobility (*a* axis) to be the 0° (or 180°) reference as well. By comparing with experimental data, the calculation results offer a qualitative explanation that for the device with polar coordinates, the direction of 0° (or 180°) approaches the *a* axis with the lowest mobility, 120° (or 300°) approaches the *b* axis with the highest mobility and 90° (or 270°) approaches the direction perpendicular to the *a* axis with a moderate mobility value between the two extremes. The quantitative discrepancies between the experimental and theoretical results imply that with continuous improvement of sample quality and material engineering, it is possible to achieve intrinsic properties that approach the real potential of ReS_2_ in high-performance device applications.

### Digital inverter based on anisotropic ReS_2_ FETs

Anisotropic ReS_2_ FET devices with expected high mobility could have important applications in future nanoscale electronics, especially on 2D logic circuits^27^,^28^,^29^,^30^,^31^ with demanding requirements of scalability (<10 nm) and large-scale integration. In contrast to conventional complementary metal-oxide-semiconductor technologies, tailoring material properties and integrated circuit design variables (such as the channel width/length ratio)^32^ to optimize circuit performance on such a rigorous scale will remain a significant challenge. In this context, the lattice orientation could be used as an alternate design variable to tune device transport properties and optimize circuit performance in future 2D integrated circuits based on anisotropic semiconducting materials.

**Figure 4:**
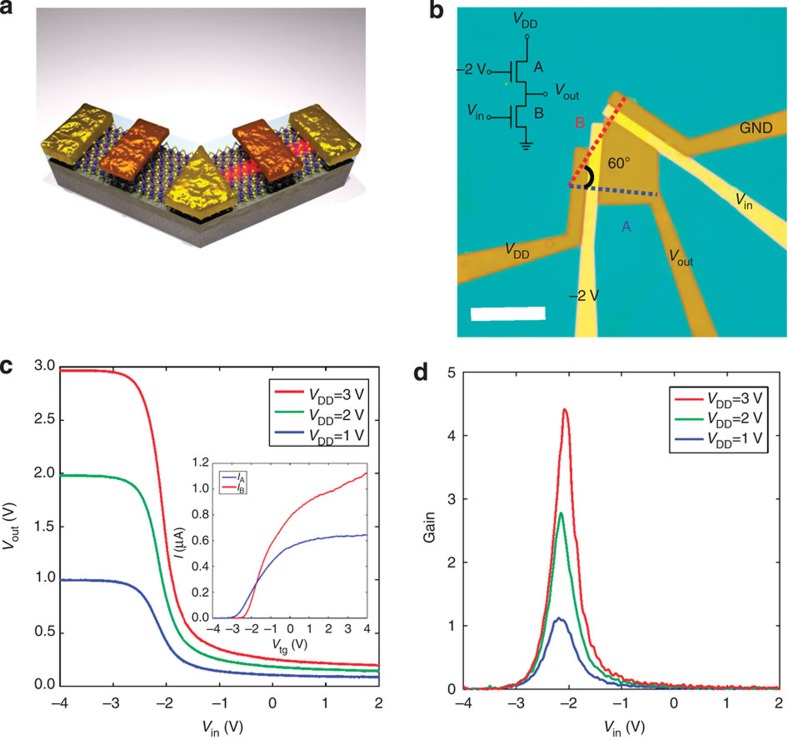
Integrated digital inverters. (**a**) A schematic showing the structure of an inverter combining two top-gated anisotropic ReS_2_ FETs. The left FET is along the *a* axis, and the right FET is along the *b* axis, where a Re atomic chain is highlighted in red. (**b**) Optical image of a typical inverter device. A quadrilateral-shaped few-layer ReS_2_ flake with a 60° inner angle was used to fabricate FETs along two axes covered by 15-nm-thick HfO_2_ as the top dielectric and two top-gate electrodes (30 nm Au). Scale bar, 10 μm. Inset: the circuit diagram of the inverter, where the top-gate voltage along the *a* axis is fixed at −2 V, the top-gate voltage along the *b* axis is the input voltage *V*_in_ and the middle shared electrode is the output voltage *V*_out_. (**c**) Transfer characteristics of an inverter operated at *V*_DD_=1, 2 and 3 V. Inset: The transfer curves of two FETs with 100 mV *V*_ds_, confirming the anisotropic behavior. (**d**) The signal gain of the inverter extracted from **c**.

As a simple example, we successfully demonstrated a ReS_2_-based prototype logic device, a 2D digital inverter. As schematically shown in Fig. 4a, the inverter can be fabricated by combining two anisotropic ReS_2_ FETs along the *a* and *b* axes (with a Re atomic chain highlighted in red). In our experiment, a quadrilateral-shaped few-layer ReS_2_ flake with a 60° inner angle was selected to fabricate two FETs along two axes. HfO_2_ was then deposited with a thickness of 15 nm as the top dielectric, followed by the fabrication of two top-gate electrodes (30 nm Au). The optical image of a typical inverter device is shown in Fig. 4b. The transfer curves along the two directions are shown in the inset of Fig. 4c, which confirms the anisotropic behaviour and determines two axes as well. The circuit diagram is shown in the inset of Fig. 4b, where the top-gate voltage on the *a* axis is fixed at −2 V, the top-gate voltage on the *b* axis is the input voltage *V*_in_ and the middle shared electrode is the output voltage *V*_out_. Figure 4c shows the voltage transfer characteristics with excellent logic-level conservation of our digital inverter, while *V*_in_ varies between −4 and 2 V for three different *V*_DD_ values (3, 2 and 1 V with different colours). When *V*_in_ is above −1 V, *V*_out_ approaches 0 V, which denotes a digital 0. Similarly, when *V*_in_ is below −3 V, *V*_out_ approaches *V*_DD_, which denotes a digital 1. The output swing (defined as the largest difference of *V*_out_) is close to the supply voltage *V*_DD_. As the most important parameter of a digital inverter, the gain is defined as |d*V*_out_/d*V*_in_|, which represents the sensitivity of *V*_out_ to the change in *V*_in_. For this device, the gain reaches 4.4 when *V*_DD_=3 V (as shown in Fig. 4d). The gain that we obtained is clearly larger than the unity gain (gain=1), which is required in integrated circuits consisting of multiple cascaded inverters, such as ring oscillators, and is comparable to the MoS_2_ based inverters^29^,^30^.

In conclusion, we have systematically studied the anisotropic properties of atomically thin ReS_2_, a semiconducting 2D TMD with a distorted 1T structure. We fabricated monolayer and few-layer ReS_2_ FET devices that exhibit competitive performance, including a high current on/off ratio (∼10^7^) and a steep subthreshold swing (100 mV per decade) at room temperature. We also obtained an anisotropic ratio along the two principle axes of ReS_2_ that is the highest obtained for all experimentally studied 2D materials. Finally, we successfully demonstrated a digital inverter device by integrating two anisotropic ReS_2_ FET devices. Our results underscore the unique properties of semiconducting 2D materials with low symmetry, which can be exploited for novel applications in future electronics.

During the preparation of this manuscript, we became aware of another work studying the FET properties of few-layer ReS_2_ (ref. 33).

## Methods

### Materials and devices

Single crystals of ReS_2_ were grown by the same Br_2_-assisted chemical vapour transport method described in ref. 17. We used a standard mechanical exfoliation method to isolate monolayer and few-layer ReS_2_ films. The number of layers can be identified using the colour interference of a 285-nm-thick SiO_2_ wafer, and further confirmed by measuring the thickness of the flakes using a Bruker Multimode 8 atomic force microscope. Micro Raman scattering experiments (Horiba-JY T64000) were carried out under ambient conditions in the backscattering geometry. The incident laser wavelength was 514.5 nm, and the power was <1 mW to minimize laser heating. Due to the limitations of the spectrometer, we only measured Raman modes above 100 cm^−1^.

A conventional electron-beam lithography process (FEI F50 with Raith pattern generation system) followed by standard electron-beam evaporation of metal electrodes (typically 5 nm Ti/50 nm Au) was used to fabricate monolayer and few-layer ReS_2_ FET devices.

### *Ab initio* calculations

Band structures for monolayer and few-layer ReS_2_ were calculated using the generalized gradient approximation Perdew–Burke–Ernzerhof function, as implemented in the VASP code (Vienna *ab initio* Simulation Package) within the density functional theory (DFT)^34^,^35^. Projector-augmented wave potentials were adopted^36^. The kinetic energy cutoff for the plane-wave basis set was 550 eV. The few-layer ReS_2_ was simulated with a vacuum layer of 15 Å in the interlayer direction to ensure negligible interaction between its periodic images. In the self-consistent calculations, the Brillouin zone integration was performed on uniform Monkhorst-Pack of 24 × 24 × 1 for few-layer ReS_2_ and 24 × 24 × 24 for bulk ReS_2_. The convergence criterion of self-consistent calculations for ionic relaxations was 10^−5^ eV between two consecutive steps. The internal coordinates and lattice constants were optimized until the atomic forces became <0.001 eV Å^−1^ and the pressures on the lattice unit cell became <0.5 kbr.

## Author contributions

E.L. and Y.F. contributed equally to this work. F.M. and H.Y. conceived the project and designed the experiments. E.L., Y.F. and Y.W. carried out the device fabrication and electrical measurements. Y.F., H.L. and X.W. carried out the DFT calculations. W.Z. performed the Raman spectroscopy measurements and analysis. Y-S.H. and C-H.H. performed the ReS_2_ single crystal growth. Z.C., L.W. and A.L. performed the HfO_2_ growth. F.M., H.Y., B.W., E.L. and Y.F. performed the data analysis and interpretation. F.M., E.L., Y.F. and H.Y. co-wrote the paper, with all authors contributing to the discussion and preparation of the manuscript.

## Additional information

**How to cite this article:** Liu, E. *et al*. Integrated digital inverters based on two-dimensional anisotropic ReS_2_ field-effect transistors. *Nat. Commun.* 6:6991 doi: 10.1038/ncomms7991 (2015).

## Supplementary Material

Supplementary Information Supplementary Figures 1-3, Supplementary Notes 1-4 and Supplementary References
