# Supplementary material for: Integrated digital inverters based on two-dimensional anisotropic ReS2 field-effect transistors
Source: Nat Commun. 2015 May 7;6:6991. doi: 10.1038/ncomms7991 (PMC4432591; doi:10.1038/ncomms7991)
Supplement: Supplementary Information — Supplementary Figures 1-3, Supplementary Notes 1-4 and Supplementary References [file ncomms7991-s1.pdf]

Supplementary Figures

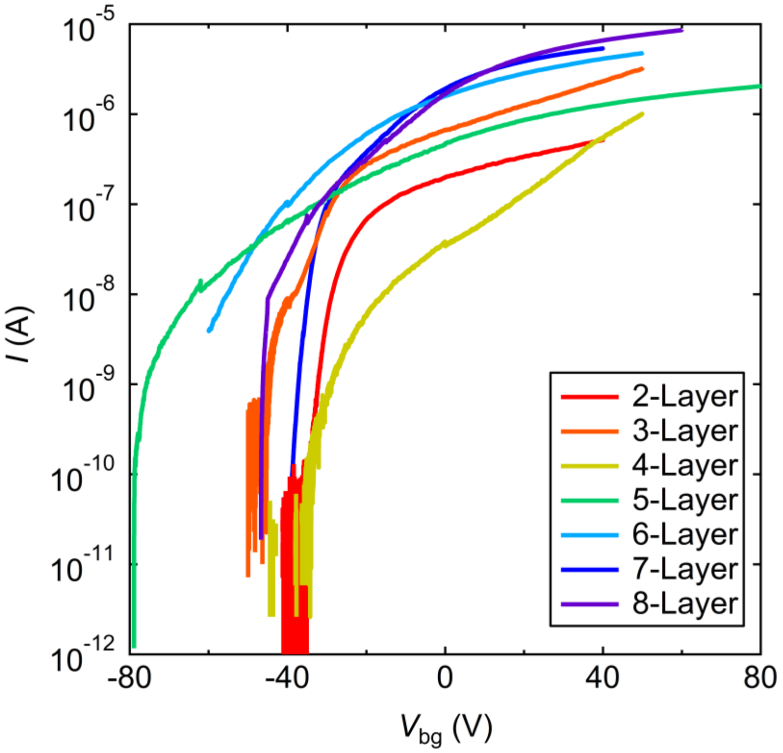

Supplementary Figure 1: Transfer curves of few-layer ReS_2 FETs. The bias voltage V_{ds} was fixed at 100 mV.

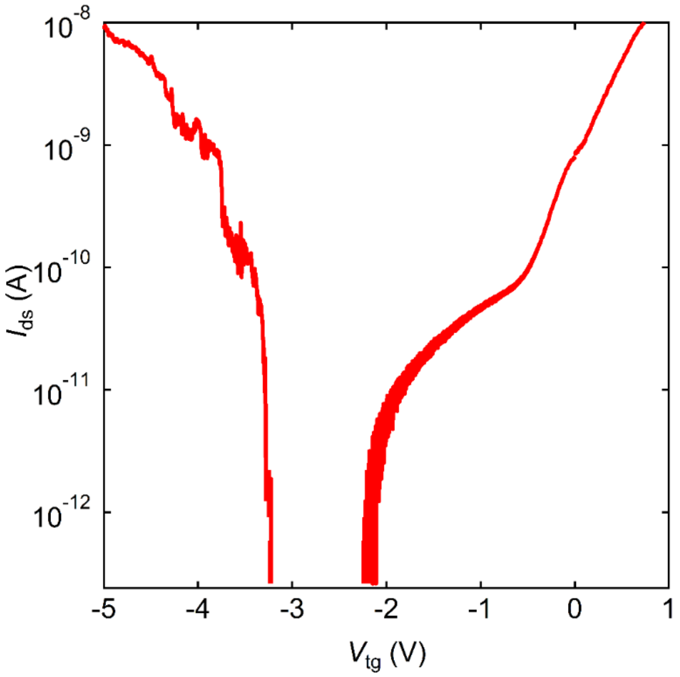

Supplementary Figure 2: Transfer curve of an ambipolar ReS_2 EDLT. The source-drain voltage was fixed at 100 mV, and the back gate remained floated. The measurements were taken at 220 K.

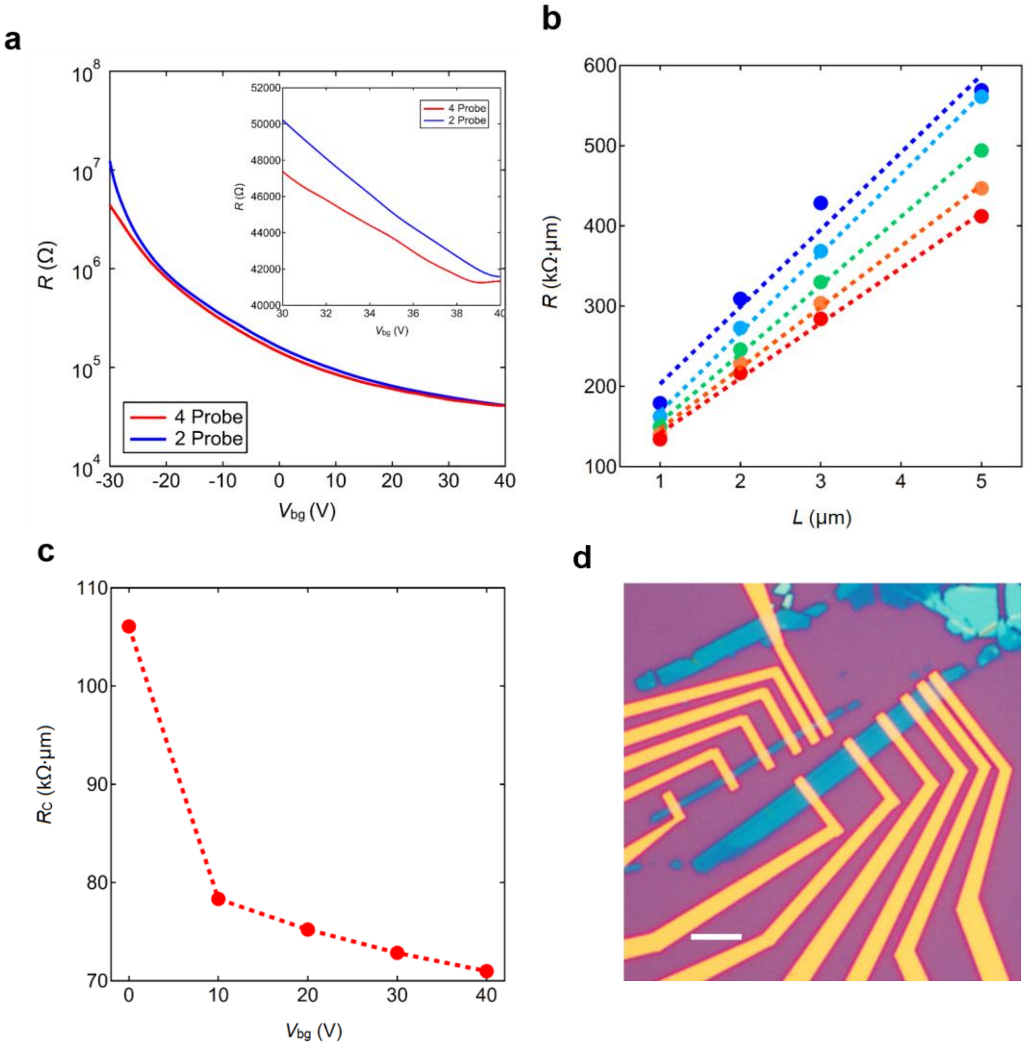

Supplementary Figure 3: Contact of ReS_2 FETs. a) Comparison of the two-probe and four-probe measurements. The inset is the zoom-in data when V_{bg} varies from 30 V to 40 V. b) The channel resistance with different length under various back gate voltage. c) The contact resistance as a function of back gate voltage. d) The microscope image of the device. The scale bar is 5 μm.

Supplementary Notes

Supplementary Note 1: Few-layer ReS_2 FETs

We measured over 40 ReS_2 FETs with thicknesses ranging from 0.8 to 7.5 nm (1-10 layers with an interlayer spacing of approximately 0.7 nm). All of the FETs behaved similarly as excellent n-type FET devices with the back gate swept between -80 V and +80 V. Some of the data from few-layer devices are summarized and shown in Supplementary Figure 1. The current on/off ratio can reach up to 10^7-10^8, which is comparable to that of MoS_2 devices [1]. All measurements were carried out in a vacuum (approximately 10^{-5} mbar) at room temperature.

Supplementary Note 2: Ambipolar behavior of ReS_2 electric double-layer transistor

To further explore the field effect of mono- and few-layer ReS_2 devices, an electric double-layer transistor (EDLT) using ionic liquid gating is introduced. In our experiments, the EDLT was fabricated by dropping a droplet of an ionic liquid, N,N-diethyl-N-(2-methoxyethyl)-N-methylammonium bis(trifluoromethylsulfonyl)imide (DEME-TFSI), onto the surface of a ReS_2 FET device. For more efficient tuning of carrier density, the side electrode pad of the ionic liquid was designed to have a larger area than the ReS_2 channel of the device. To avoid possible chemical reactions between the ionic liquid and ReS_2, all measurements were performed at 220K and in a vacuum environment of approximately 10^{-5} mbar. An ambipolar behavior was observed when we swept the ionic liquid gate voltage (V_{LG}), with a typical transfer curve measured from a seven-layer ReS_2 device shown in Supplementary Figure 2. When V_{LG} is above -2 V, the device behaves as an n-type transistor. When V_{LG} is below -3.2 V, a p-type transistor behavior appears, indicating the shift of Fermi-level E_F to access the valence band.

Supplementary Note 3: Contact of ReS_2 FETs

During the fabrication of mono- and few-layer ReS_2 FETs, we used 5 nm Ti covered by 50 nm Au as electrodes to make the contact. These devices have shown good contact behavior. To estimate the influence of contact resistance, we compared the measurement results obtained from both two-probe and four-probe methods. As shown in Supplementary Figure 3a, the resistance measured by the two-probe method (R_{2P}) is slightly larger than that of the four-probe method (R_{4P}). The resistance difference (ΔR) of R_{2P} and R_{4P} should be mainly attributed to the contact resistance. If we define $\alpha = \frac{\Delta R}{R_{2P}}$ as the weight of ΔR in R_{2P}, we found α is highly gate-tunable. In the n-doped regime as our work focused on, α is less than 10% (when V_{bg} = -20 V) and decreases notably as the doping level increases, to be as low as 1% when V_{bg} = 40 V.

We also used the transfer length method to estimate the contact resistance of the ReS_2 FETs. As shown in Supplementary Figure 3b, we measured the channel resistance with different length under various back gate voltage V_{bg}. The extrapolated contact resistance R_C versus V_{bg} is shown in Supplementary Figure 3c. The contact resistance decreases with the increasing V_{bg}, ranging from 71 kΩ to 106 kΩ, which is similar to the results reported [2]. Supplementary Figure 3d shows the optical image of the multi-terminal device.

Supplementary Note 4: Carrier mobility calculation

Our carrier mobility calculation was performed using the VASP (Vienna ab-initio Simulation Package) code [3, 4]. The results presented in the following were obtained by using the generalized gradient approximation (GGA)-Perdew-Becke-Erzenhof (PBE) function [5], a 10×5×1 mesh for the Brillouin zone sampling and a cut-off of 500 eV for the plane-wave basis set. An orthogonal supercell was created for ReS_2 sheets, with the atomic plane and its neighboring image separated by a 36 Å vacuum layer. All of the structures were relaxed until the Hellmann−Feynman forces became less than 0.01 eV$Å^{-1}$.

In 2D materials, the carrier mobility is given by the following formula [6, 7]:

$$\mu_{2D} = \frac{2e_0\hbar^3 C_q}{3k_B T E_1^2 m^{*2}} \qquad (1)$$

where m^* is the effective mass and T is the temperature (set to be 300K in our calculation). The term E_1 is the deformation potential constant, which denotes the shift in the band edges along the transport directions induced by strain ε (calculated using a step of 1%). It is defined as $E_1 = \frac{\partial E_M}{\partial \varepsilon}$, where E_M is the energy of the conduction band minimum for electrons and the valence band maximum for holes. C_q is the stretching modulus caused by ε, which can be calculated by $C_q = \frac{1}{S_0}\frac{\partial^2 E_{\text{total}}}{\partial \varepsilon^2}$, where E_{total} is the total energy and S_0 is the lattice volume at equilibrium for a 2D system.

Supplementary References

1. Radisavljevic, B. *et al*. Single-layer MoS2 transistors. *Nat. Nanotech.* **6**, 147-150 (2011).

2. Corbet, C. M.; McClellan, C.; Rai, A.; Sonde, S. S.; Tutuc, E.; Banerjee, S. K. Field effect transistors with current saturation and voltage gain in ultrathin ReS_2. *ACS Nano* **9**, 363-370 (2015).

3. Hafner, J. & Kresse, G. *Ab initio* molecular dynamics for liquid metals. *Phys. Rev. B* **47**, 558-561 (1993).

4. Kresse, G. & Furthmuller, J. Efficient iterative schemes for *ab initio* total-energy calculations using a plane-wave basis set. *Phys. Rev. B* **54**, 11169-11186 (1996).

5. Perdew, J.P., Burke, K. & Ernzerhof, M. Generalized gradient approximation made simple. *Phys. Rev. Lett*. **77**, 3865-3868 (1996).

6. Price, P.J. Two-dimensional electron transport in semiconductor layers. I. Phonon scattering. *Ann. Phys*. **133**, 217-239 (1981).

7. Xi, J.Y. *et al*. First-principles prediction of charge mobility in carbon and organic nanomaterials. *Nanoscale* **4**, 4348-4369 (2012).
